# Supplementary material for: A hyperconformal dual-modal metaskin for well-defined and high-precision contextual interactions
Source: Nat Commun. 2025 Nov 26;16:10573. doi: 10.1038/s41467-025-65624-z (PMC12658143; doi:10.1038/s41467-025-65624-z)
Supplement: Supplementary file 1 — Supplementary Information [file 41467_2025_65624_MOESM1_ESM.pdf]

# Supplementary Information

# A hyperconformal dual-modal metaskin for well-defined and high-precision contextual interactions

*Shifan Yu[1], Zhenzhou Ji[1], Lei Liu[1], Zijian Huang[1], Yanhao Luo[1], Huasen Wang[1], Ruize Wangyuan[1], Ziquan Guo[1], Zhong Chen[1], Qingliang Liao[2,3], Yuanjin Zheng[4], Xinqin Liao[1]**

[1] Department of Electronic Science, Xiamen University, Xiamen 361005, China

[2] Academy for Advanced Interdisciplinary Science and Technology, Key Laboratory of Advanced Materials and Devices for Post-Moore Chips Ministry of Education, University of Science and Technology Beijing, Beijing 100083, China

[3] Beijing Key Laboratory for Advanced Energy Materials and Technologies, School of Materials Science and Engineering, University of Science and Technology Beijing, Beijing 100083, China

[4] School of Electrical and Electronic Engineering, Nanyang Technological University, Singapore 639798, Singapore

*E-mail: liaoxinqin@xmu.edu.cn

## Note S1. Relative position calculation of touch points

The decoupling of the proprioceptive stretch signal and exteroceptive touch position signal is facilitated by the distinct patterns they exhibit. The stretch signal and touch position signal can each be modeled as linear changes in resistance. By characterizing the signal variations under specific stretching conditions, it becomes possible to accurately decouple and determine the relative touch position. To determine the relative touch position in a stretched state, the resistance change of the hyperconformal dual-modal (HDM) metaskin is converted into a corresponding voltage signal within a controlled voltage range via a voltage divider module. By accurately measuring the voltage across the constant resistor in the voltage divider, the relative touch position can be computed as detailed below.

For a circuit composed of a constant resistor $R_c$ and a stretchable resistor $R_s$, the voltage across the constant resistor is determined by the voltage divider rule:

$$V_{\mathrm{c}} = V \cdot \frac{R_c}{R_c + R_s} \tag{1}$$

When no touch occurs, the readout voltage $V_r = V_c$ is influenced by the resistance values in the circuit. Since the constant resistor $R_c$ is fixed, the voltage across it can be directly determined by the value of the stretchable resistor $R_s$. Thus, the touch voltage is given by:

$$V_{c,0} = V \cdot \frac{R_c}{R_c + R_{s,0}} \tag{2}$$

$$V_{c,t} = V \cdot \frac{R_c}{R_c + R_{s,t}} \tag{3}$$

The relative touch position is defined as the ratio of the decrease in resistance due to the touch to the total resistance of the stretchable element:

$$P_t = \frac{R_{s,0} - R_{s,t}}{R_{s,0}} \tag{4}$$

Substituting $R_s$ and $R_{s,t}$ into the position equation, we get:

$$P_t = \frac{\left(\frac{V \cdot R_c}{V_{c,0}} - R_c\right) - \left(\frac{V \cdot R_c}{V_{c,t}} - R_c\right)}{\frac{V \cdot R_c}{V_{c,0}} - R_c} \tag{5}$$

The final expression for the relative position of the touch point is:

$$P_t = \frac{V \cdot (V_{c,t} - V_{c,0})}{V_{c,t} \cdot (V - V_{c,0})} \tag{6}$$

## Note S2. The surface energy correlation

During the spin-coating process of WPU on a hydrophobic substrate, differences in wettability made it challenging for the WPU solution to spread uniformly. High-concentration WPU solutions exhibited resistance to self-contraction, forming a more uniform film across the substrate, while low-concentration solutions showed a tendency to retract into smaller droplets. This phenomenon can be explained in terms of liquid-gas surface tension. According to the Gibbs adsorption equation:

$$\frac{d\gamma_{LG}}{dC} = -RT\Gamma \tag{7}$$

Where, $C$ is the concentration of the solute, $R$ is the gas constant, $T$ is the temperature, $\Gamma$ represents the surface excess concentration of the solution. $\gamma_{LG}$ is the interfacial tension between the hydrophobic substrate and gas phase. It can be inferred that as the solute concentration increases, solute molecules accumulate at the liquid-gas interface ($\Gamma > 0$), leading to a reduction in $\gamma_{LG}$. According to Young's equation:

$$\cos\theta = \frac{\gamma_{SG} - \gamma_{SL}}{\gamma_{LG}} \tag{8}$$

Where, $\theta$ is the contact angle between the liquid and the solid. For low-concentration WPU solutions, the $\gamma_{LG}$ is relatively high. Thus, after spin-coating dilute solutions, the contact angle on the hydrophobic surface tends to be large. At this point, the liquid surface undergoes spontaneous contraction due to a Young-Laplace forces imbalance. In contrast, high-concentration waterborne polyurethane solutions, due to the reduction in liquid-gas surface tension and increased viscosity, exhibit greater resistance to self-contraction, enabling a more uniform coating on the hydrophobic substrate.

## Note S3 Method of feature extraction

To extract relevant features from the sensor input domain, four distinct feature extraction methods are employed. These methods provide diverse signal characteristics, enhancing the model's ability to learn from complex temporal patterns.

(1) Derivative

The derivatives of the sensor signals are computed to capture the rate of change and acceleration of the input data. Let $s_i$ denote the sensor signal at time $i$. The first derivative is defined as:

$$s_i' = \frac{\partial s_i}{\partial t} \tag{9}$$

and the second derivative is computed as:

$$s_i'' = \frac{\partial^2 s_i}{\partial t^2} \tag{10}$$

To ensure consistency across varying signal magnitudes, these derivative features are standardized using z-score normalization, represented as:

$$s_{\text{norm}} = \frac{s - \mu}{\sigma} \tag{11}$$

where $\mu$ and $\sigma$ are the mean and standard deviation of the respective feature.

(2) Signal energy features

The energy of the sensor signal at each time point is calculated as the squared value of the signal:

$$E_i = s_i^2 \tag{12}$$

To capture local variations, a sliding window of size $w$ is used to compute the mean energy across the window, yielding the rolling energy:

$$E(t) = \frac{1}{w} \sum_{i=t-w}^{t} E_i \tag{13}$$

(3) Hilbert transform

The Hilbert transform is applied to the sensor signals to derive additional temporal characteristics, resulting in an analytic signal $a(t)$ of the form:

$$a(t) = s(t) + j \cdot H(s(t)) \tag{14}$$

where $H(s(t))$ represents the Hilbert transform of the original signal. From this analytic representation, the instantaneous amplitude $A(t)$ and instantaneous phase $\phi(t)$ are extracted as follows:

$$A(t) = |a(t)| \tag{15}$$

$$\phi(t) = unwrap(\arg(a(t))) \tag{16}$$

(4) Temporal difference features

Temporal differences are employed to quantify changes in the signal over time. The first-order and second-order differences are computed as:

$$\Delta s_i^{(1)} = s_i - s_{i-1} \tag{17}$$

$$\Delta s_i^{(2)} = s_i - 2s_{i-1} + s_{i-2} \tag{18}$$

As described, the combination of derivative features, signal energy, Hilbert transform, and temporal differences creates a rich feature set that encapsulates both instantaneous and temporal properties of the sensor signals. This enhances the explanation of the model's dynamic input space, increasing data complexity to facilitate deeper analysis.

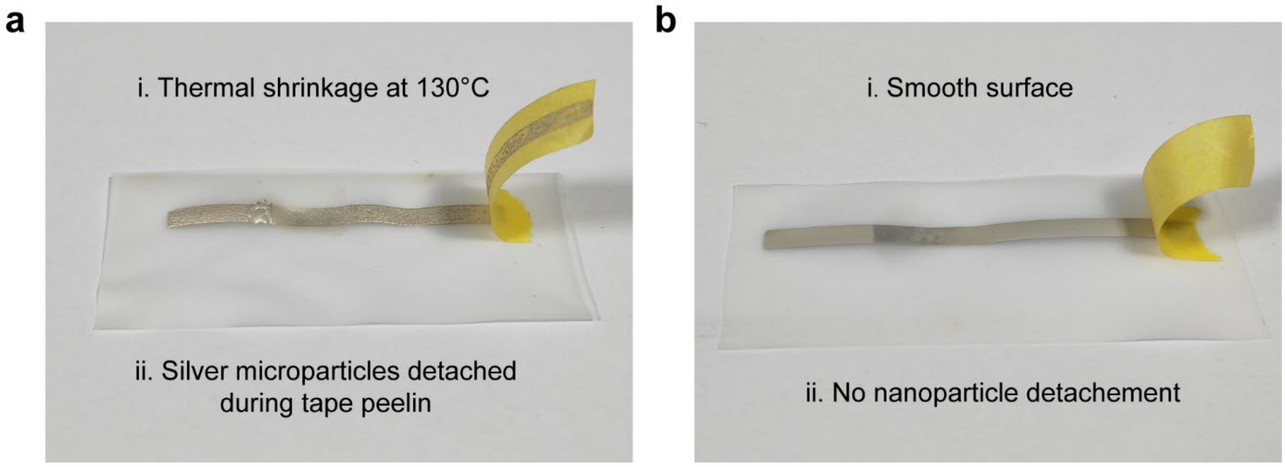


**Fig. S1. Comparison of hot-sintered Ag path and water-based Ag path.**

(a) Performance of hot-sintered Ag path on Thermoplastic Polyurethane (TPU film. The sintering temperature of conventional Ag aggregation is at least 130°C, which can induce detrimental shrinkage, posing even irreversible electromechanical damage on thin film. Besides, the bonding strength between silver microparticles is relatively weak, causing detachment easily under tape peeling forces or even slight friction, making it unsuitable for superficial electronics. (b) Performance of water-based Ag path on TPU film. In contrast, water-based Ag nanocomposite only requires moderate curing temperature and maintain strong interfacial bonding strength with the substrate. All these provide superior resistance to peeling and friction.

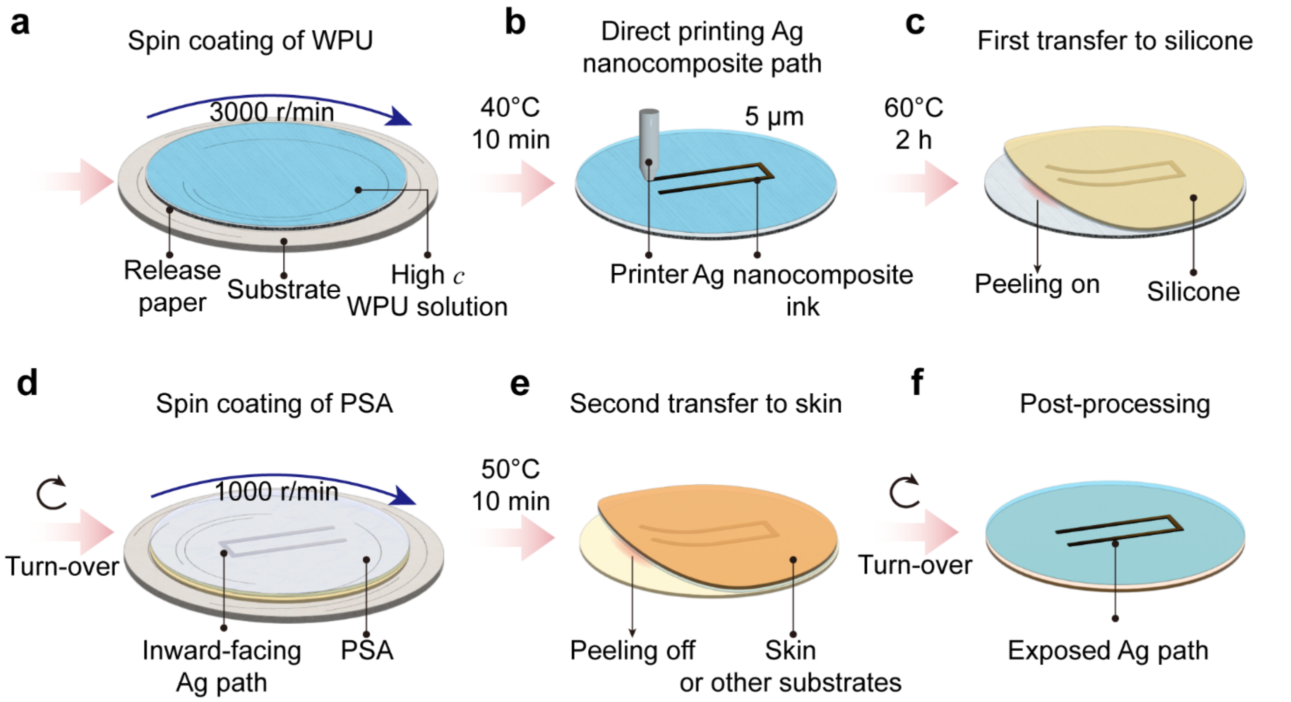


**Fig. S2. Fabrication process of WPU film with Ag nanocomposite paths.**

(a) Spin coating of WPU solution onto release paper at a spinning speed of 4000 rpm, followed by drying at 40°C for 10 minutes. (b) Direct printing of Ag nanocomposite paths, followed by drying at 60°C for 30 minutes. (c) First transfer of the WPU film to a silicone substrate through peeling. (d) Spin coating of PSA after turning the WPU film over, with the Ag path facing inward, followed by drying at 50°C for 10 minutes. (e) Second transfer of the WPU film to the skin or another substrate, leveraging pressure sensitive adhesive (PSA) for adhesion. (f) Process completed, with the Ag path exposed on the outer side of the film, ready for subsequent circuit connection and peripheral processing.

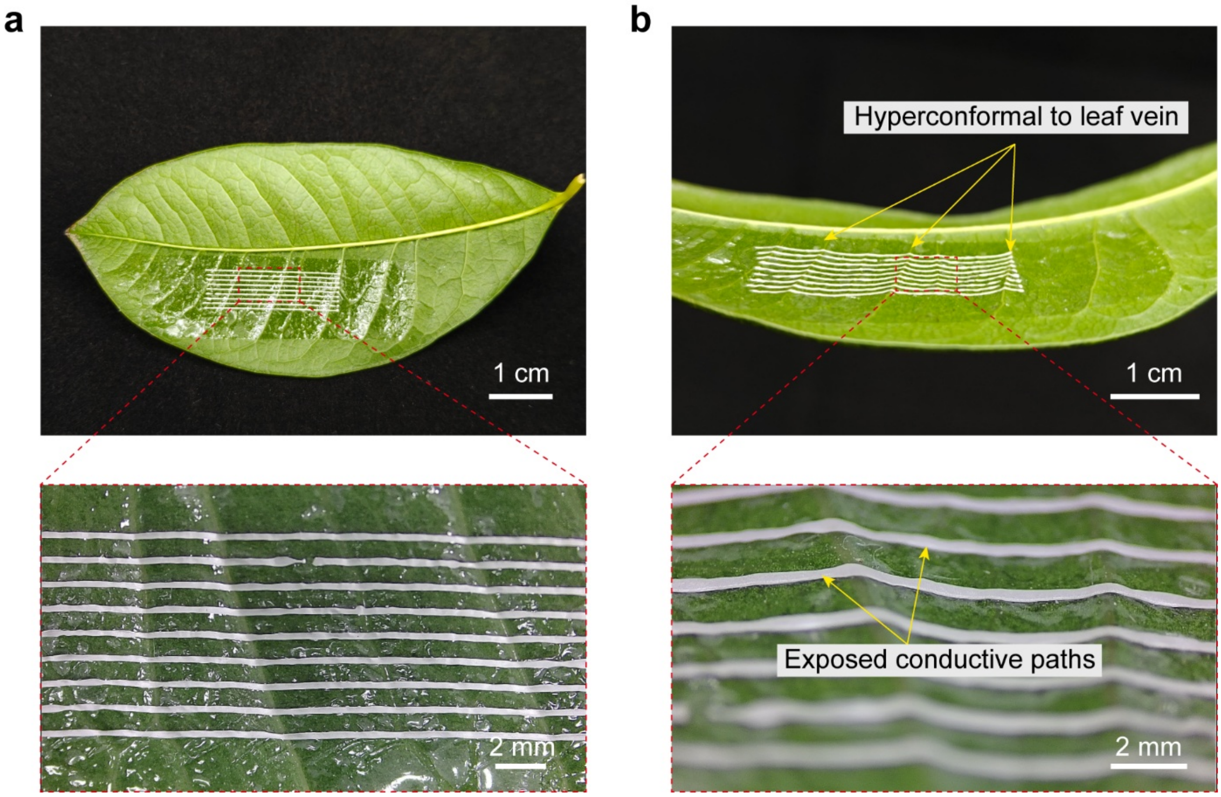


**Fig. S3. Demonstration of conformal integration of the HDM metaskin on a leaf surface.**

(a-b) Seamless conformance of the printed conductive paths to the leaf veins. Magnified views highlight the exposed conductive paths with precise alignment to the complex surface topography.

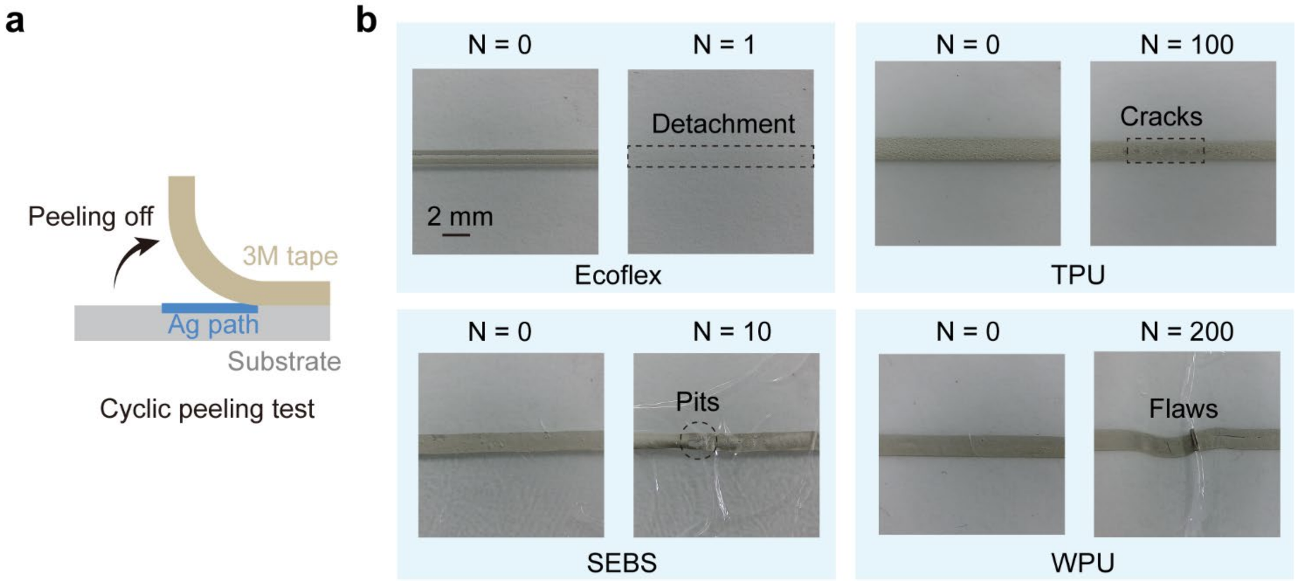


**Fig. S4. Adhesion testing of printed Ag nanocomposite paths on various substrates.**

(a) Schematic of the striping test setup, where 3M tape is repeatedly applied and stripped to evaluate the adhesion strength of Ag nanocomposite conductive paths. (b) Results for different substrates (Ecoflex, TPU, SEBS, and WPU) at various triping cycles. Ecoflex shows complete detachment after 1 cycle. SEBS exhibits large-area damage after 10 cycles. TPU develops cracks after 100 cycles. WPU demonstrates the best performance, with only subtle damage after 200 cycles.

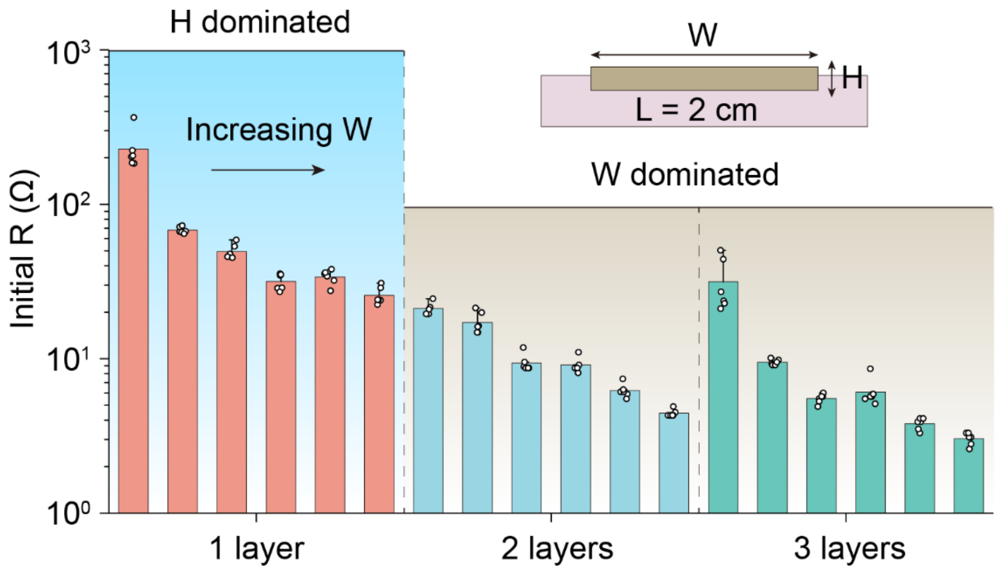


**Fig. S5. Relationship between printing parameters and the conductivity of printed Ag nanocomposite paths.**

In the 1-layer configuration, the initial resistance is dominated by thickness (*H*), resulting in higher resistance. For 2-layer and 3-layer configurations, the resistance becomes width-dominated (*W*), decreasing as the line width increases. *W* represents the line width (ranging from 0.25 mm to 1.5 mm), and *H* corresponds to the printing thickness (1-layer, 2-layer, and 3-layer configurations). The measured length of *L* is fixed at 2 cm. (Data are mean ± SD, N = 6, unit: Ω)

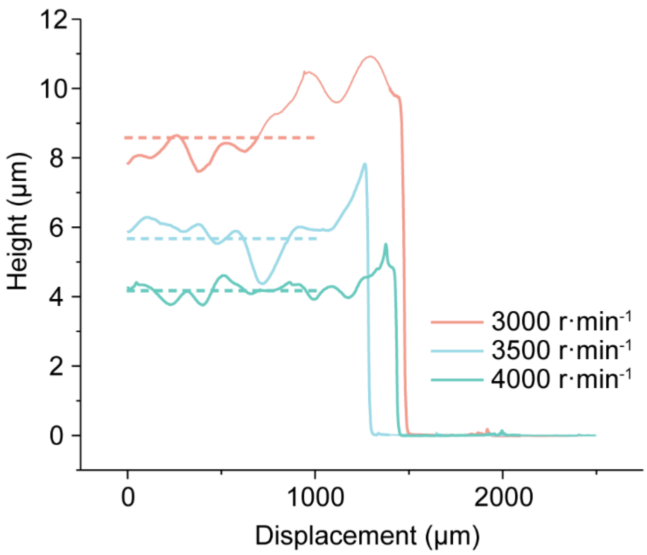


**Fig. S6. Thickness profiles of films at different spin-coating speeds.**

Decrease in film thickness with increasing spin speed. Dashed lines indicate the average film thickness for each spin speed, with a thickness of only 4 μm at a spin-coating speed of 4000 r·min$^{-1}$.

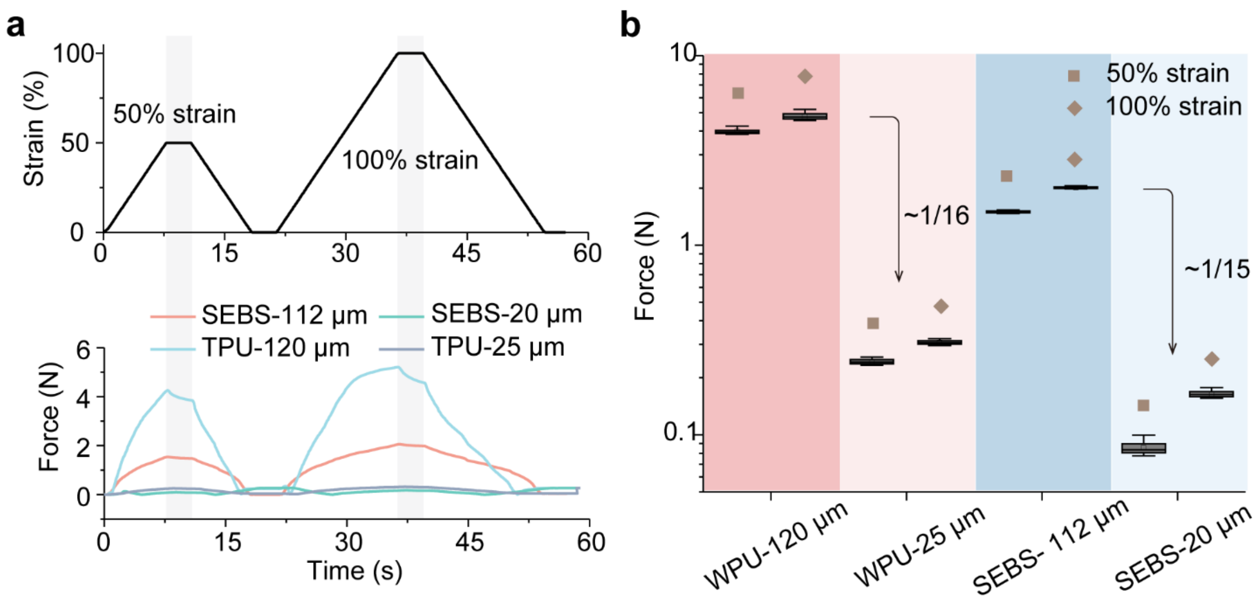


**Fig. S7. Force comparison for stretching films of varying thickness, highlighting the perceptible stress level of the film on human skin.**

(a) Strain-force relationship during cyclic stretching at 50% and 100% strain levels for films with different thicknesses (SEBS-112 μm, SEBS-20 μm, WPU-120 μm, WPU-25 μm). Thinner films require significantly less force for the same strain. Note: Stretching forces for films thinner than 20 μm are challenging to measure accurately with instruments; therefore, the film thickness is set to approximately 20 μm. (b) A quantitative comparison showing that reducing film thickness to one-quarter lowers the required force by approximately one-sixteenth, enabling nearly imperceptible interaction on human skin. (single test, N > 50, unit: N)

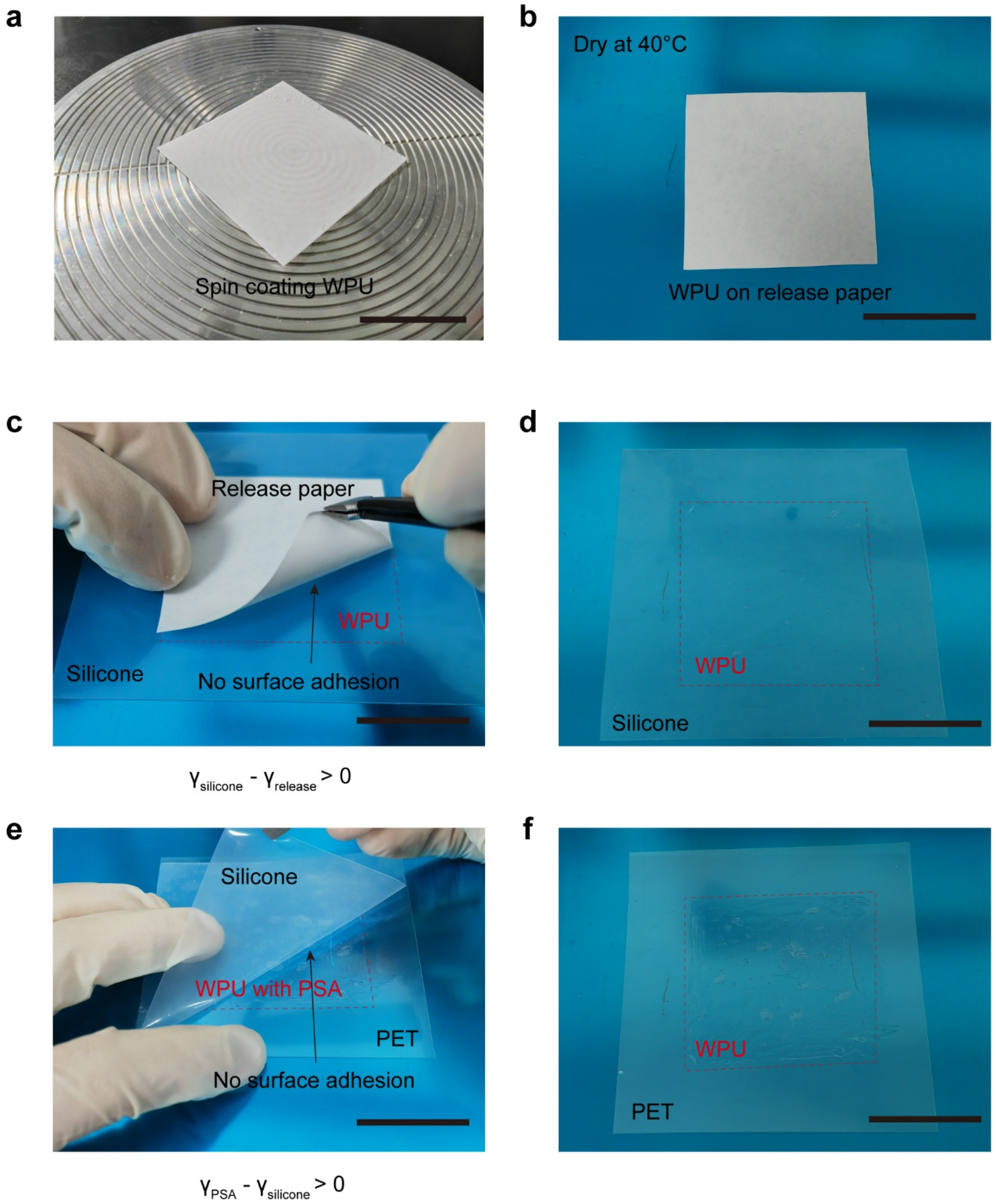


**Fig. S8. Two-step transfer process of WPU films.**

(a) Spin-coating of WPU onto release paper. (b) Low-temperature hot drying of the WPU layer, with the exposed side designated as the front side. (c) Peeling the WPU film off the release paper without surface adhesion to the silicone. (d) Transfer of the WPU film onto a silicone substrate, exposing the back side of the film. (e) Transfer of the WPU film to a PET substrate using a highly viscous PSA. The low surface energy of silicone allows the film to be stripped without inducing local strain. (f) Final WPU layer on the PET substrate, with the front side exposed. Scale bar: 2cm.

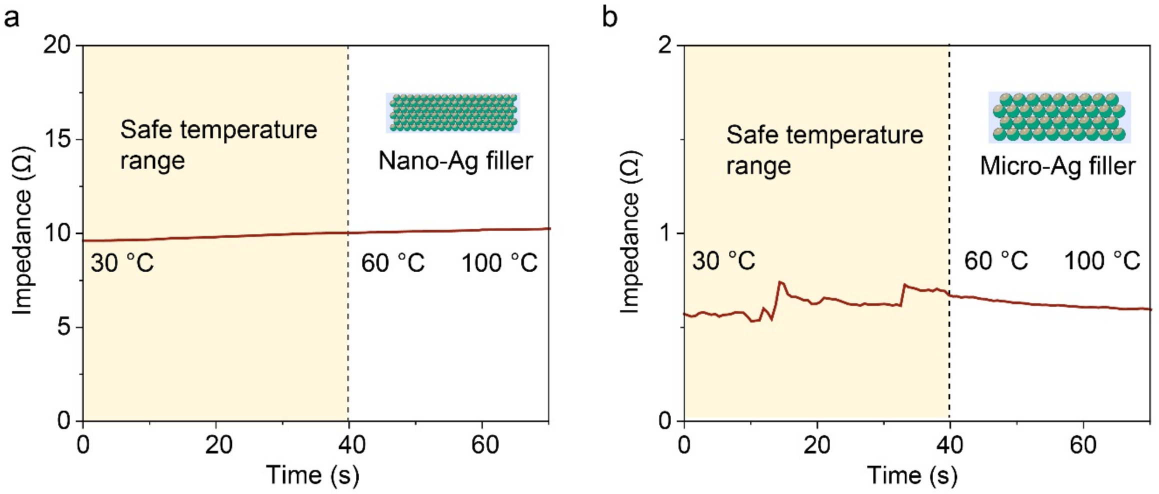


**Fig. S9. Impedance stability comparison in the 30-100°C range for (a) nano-Ag and (b) micro-Ag filled composites.**

Thermal stability of Ag-filled conductive composites was investigated through a continuous heating test. Both nano- and micro-Ag composites exhibit significantly suppressed positive temperature coefficient (PTC) effects compared to pure metals due to their discontinuous conductive medium. While the nano-Ag composite demonstrated exceptional stability with negligible impedance variation (about 4% in the safe temperature range from 30°C to 60°C), the micro-Ag composite showed greater temperature sensitivity, displaying measurable fluctuations even below 60°C. The superior performance of nano-Ag composite suggests their densely packed networks are more resistant to potential thermal expansion and particle rearrangement.

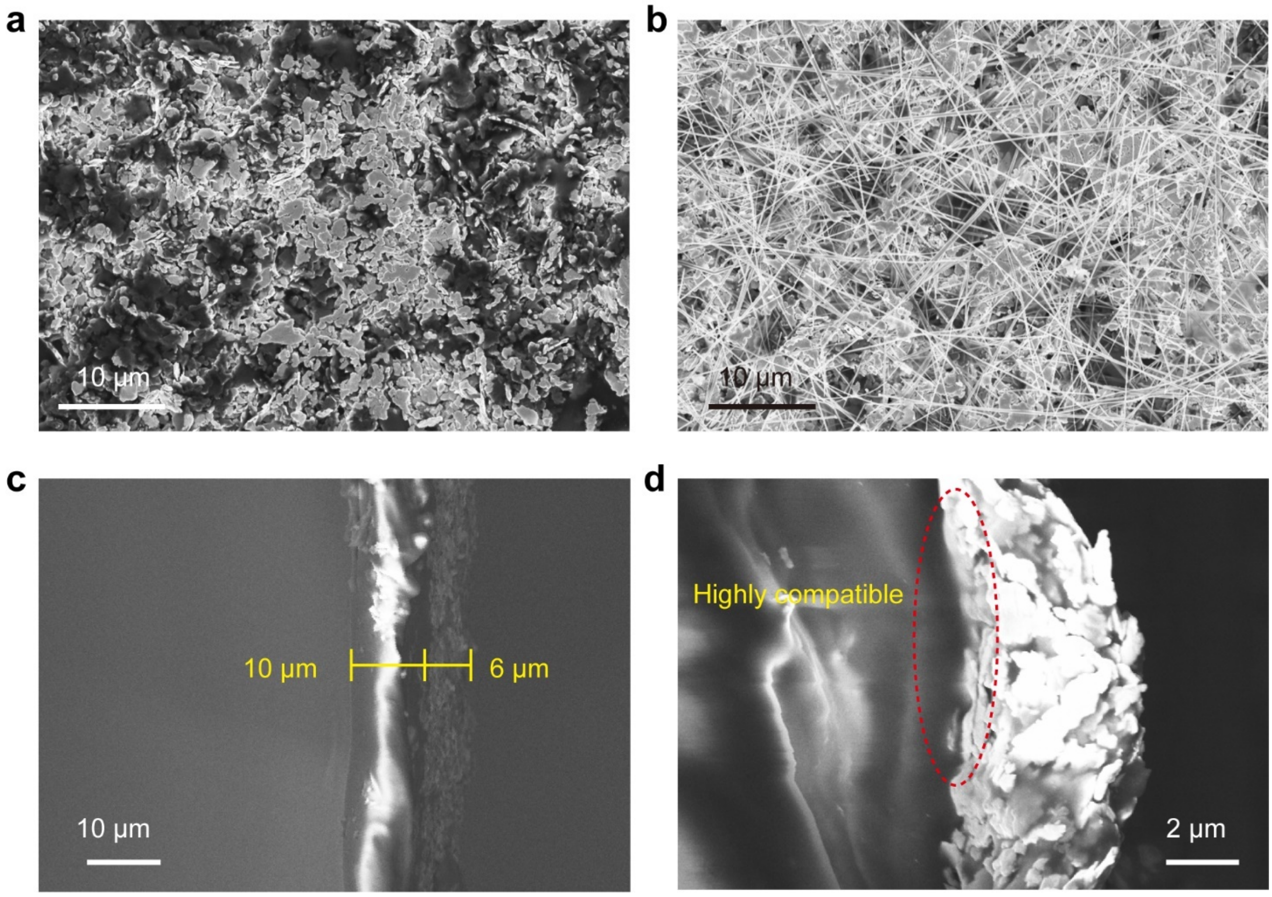


**Fig. S10. SEM image of Ag aggregates and the HDM metaskin.**

(a-b) Comparison of Ag NP aggregates and Ag NP/Ag NW nanocomposite. Under the influence of the PU binder, Ag NPs are tightly bonded and uniformly dispersed. In contrast, the Ag NP/Ag NW nanocomposite forms an interpenetrating network. This reinforced conductive network effectively mitigates crack-induced failures and yields more stable strain-resistance characteristics. (c–d) Cross-sectional views of the HDM metaskin film. The measured average thickness of the film substrate is only 10 μm, and the conductive layer has an average thickness of 6 μm. Furthermore, due to the high compatibility between the substrate film and the binder, a strong interfacial embedding of Ag NPs can be observed.

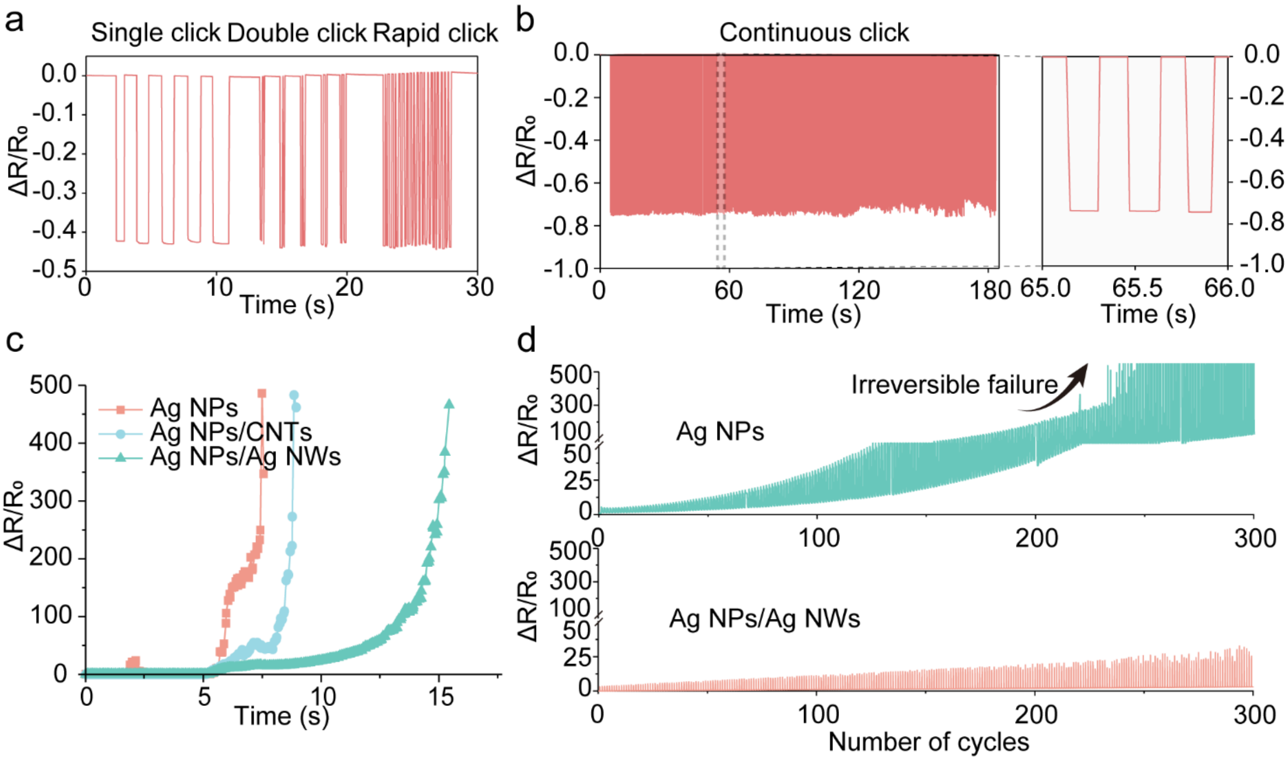


**Fig. S11. Touch and stretch signal stability of the HDM metaskin.**

(a) Signal responses for various touch events, including single-click, double-click, and rapid clicks. (b) Stability test of the HDM metaskin under continuous touch at a frequency of 3 Hz. (c) Tensile resistance response curve of sensitive paths (path length: 35 mm; stretching speed: 1 mm/s). Results indicate that Ag NP and Ag NP/CNT paths experience conductive failure at an early stage, while the Ag NP/Ag NW path exhibits a smooth increase during stretching. (d) Cyclic stability of sensitive paths printed with Ag NPs and Ag NPs/Ag NWs under 10% cyclic stretching (path length: 35 mm; path width: 3 mm). The Ag NP path shows conductivity degradation due to irreversible crack formation, whereas the Ag NP/Ag NW nanocomposite network facilitates crack recovery, demonstrating superior stability.

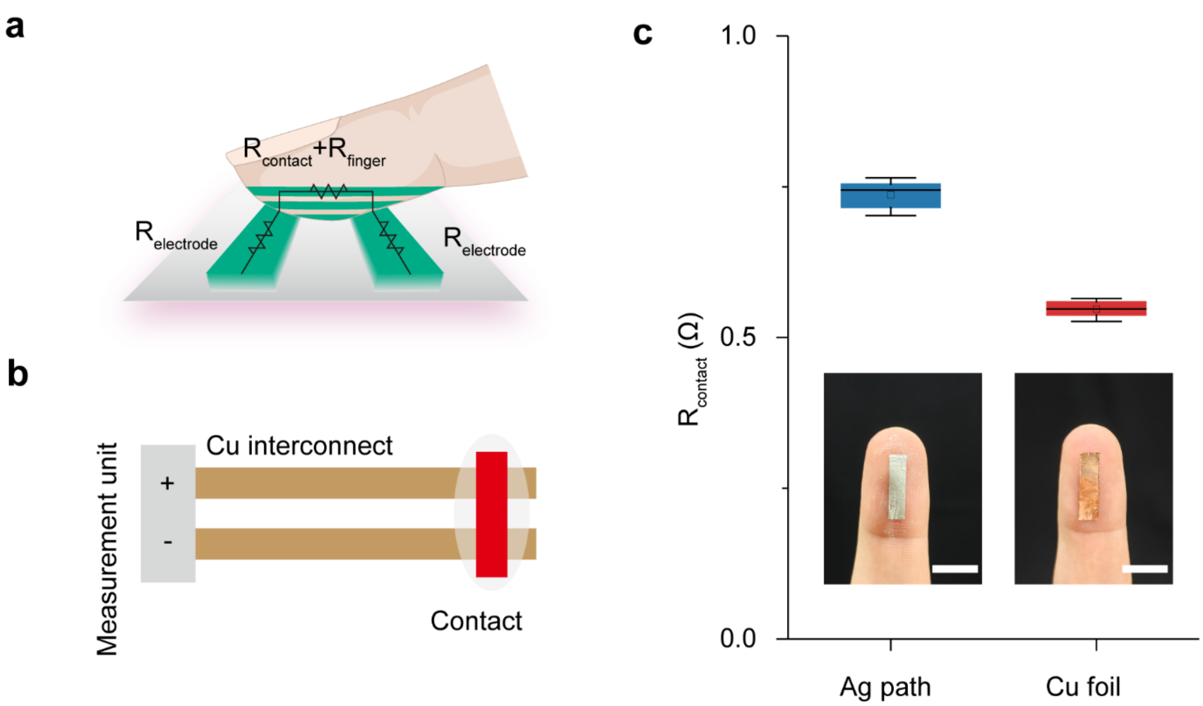


**Fig. S12. Contact resistance of finger trigger in exteroceptive mode.**

(a) Principle of exteroceptive triggering: The HDM metaskin trigger on the fingertip short-circuits parallel conductive pathways. The total circuit resistance is expressed as $R_{\text{total}} = 2R_{\text{electrode}} + R_{\text{contact}} + R_{\text{finger}}$, where the contact resistance at the fingertip directly influences the overall positioning calculation. (b) Measurement setup using copper interconnects to evaluate $R_{\text{contact}} + R_{\text{finger}}$. (c) Comparison of contact resistance between the HDM metaskin device and copper foil. The metaskin exhibits low contact resistance as a finger trigger, enabling it to effectively function as a trigger by simulating a short circuit in parallel conductive pathways. Additionally, the metaskin adheres conformally to the fingertip, making it imperceptible and ensuring stable performance. Scale bar: 1 cm. (Data are mean ± SD, N = 3)

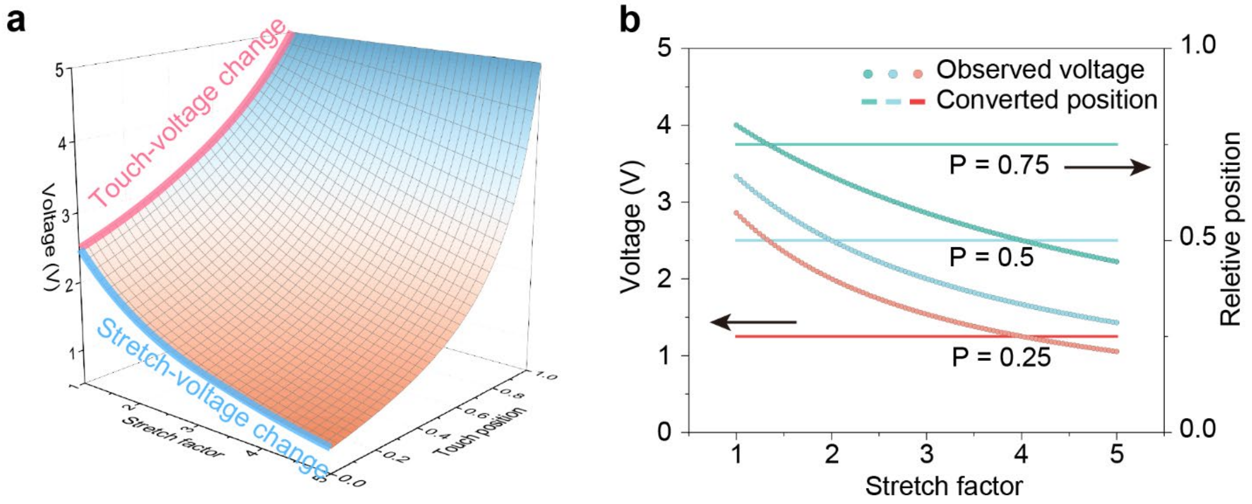


**Fig. S13. Simulated calculation of touch position under varying stretch factors.**

(a) Continuous simulation illustrating the relationship between stretch factor, touch position, and voltage. (b) Simulated calculations at three distinct positions under different stretch factors. The results indicate that while voltage decreases with increasing stretch, ideally, the calculated touch position remains unaffected.

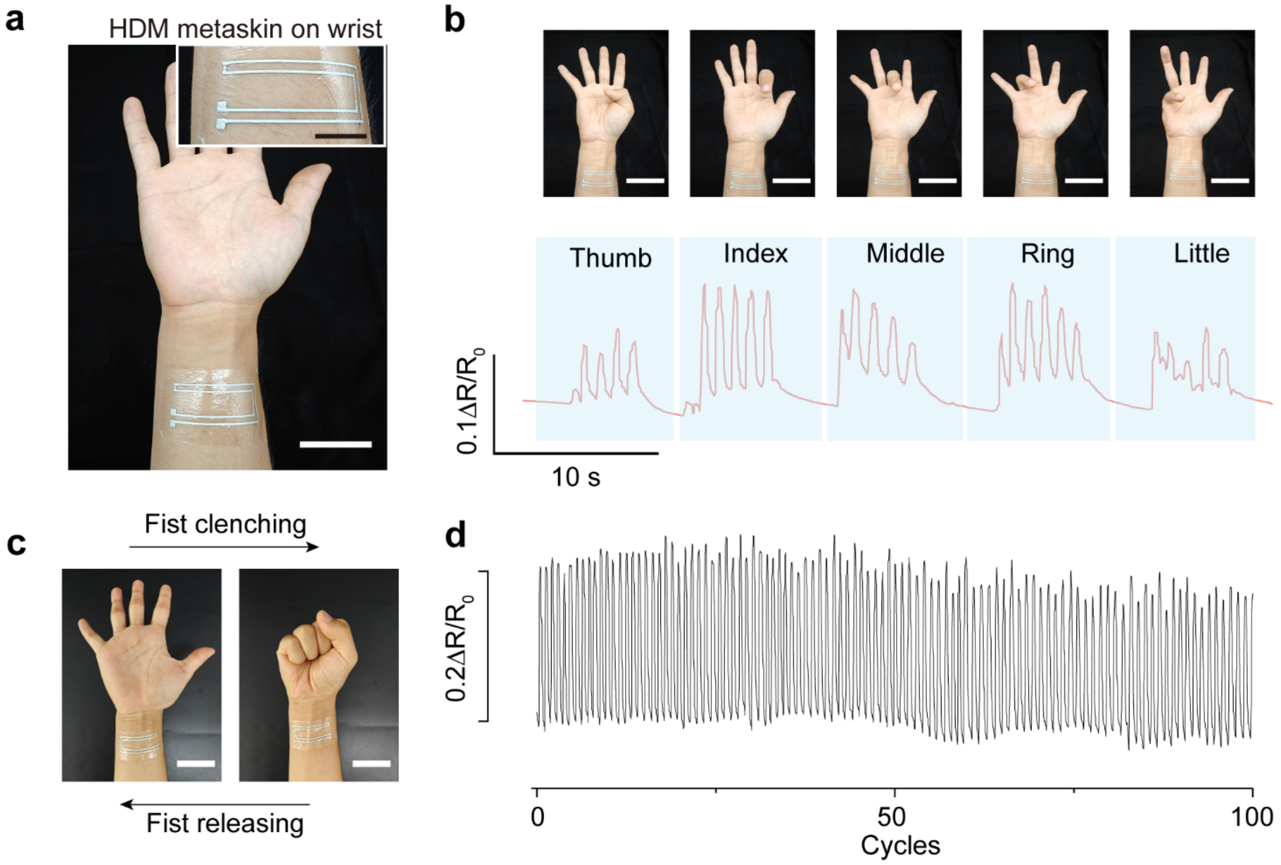


**Fig. S14. Real-time monitoring of hand and wrist motions using the HDM metaskin through detection of fine strain signals from the wrist flexor muscles.**

(a) Conformal attachment of the HDM metaskin to the volar wrist for sensing. Scale bar: 5 cm (main view) and 2 cm (inset). (b) High sensitivity of the device, enabling the detection of subtle strains of the wrist flexor muscles associated with the movements of five individual fingers. Scale bar: 5 cm. (c) Cyclic fist clenching and releasing captured by the metaskin. Scale bar: 5 cm. (d) Signal stability over 100 cycles of fist clenching and releasing, showing durability and consistency of the sensor's performance.

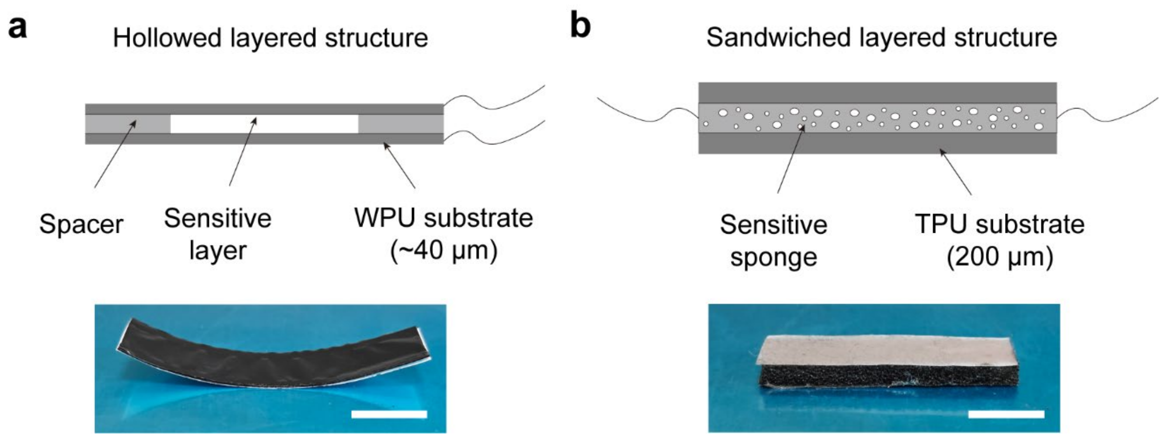


**Fig. S15. Structures of layered sensing electronics.**

(a) Hollowed structured devices consisting of a spacer, a sensitive layer, and a WPU substrate (40 μm). Scale bar: 1 cm. (b) Sandwiched structured devices composed of a sensitive sponge layer and a TPU substrate (200 μm). Scale bar: 1 cm.

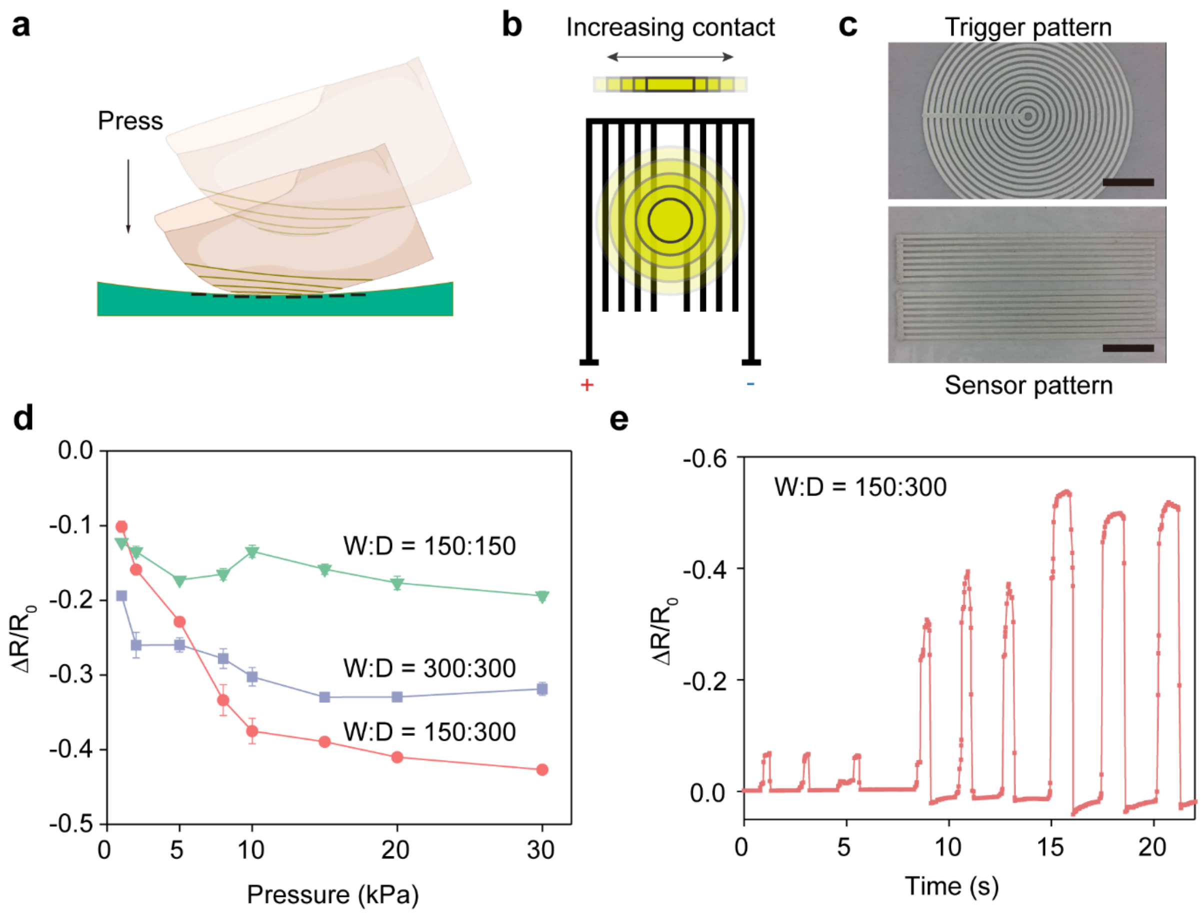


**Fig. S16. Touch pressure response using two-dimensional surface configurations.**

(a) Schematic of the sensor under pressing action, showing the gradual increase in contact area with pressure. (b) Illustration of the pressure-sensitive sensor with a stripe-shaped structure, where increasing contact improves the conductivity. (c) Microscopic images of pressure-sensitive sensor and ring-shaped trigger. Scale bar: 5 mm. (d) Resistance changes as a function of applied pressure (0–30 kPa) for different width-to-distance (W:D) ratios (unit: μm). When the W:D ratio of the sensor is 150:300, the output signal range is larger, enabling a more accurate mapping of pressure variations. (Data are mean ± SD, N = 3) (e) Signal response of the pressure-sensitive sensor with different pressure intensities of light press, medium press, and heavy press.

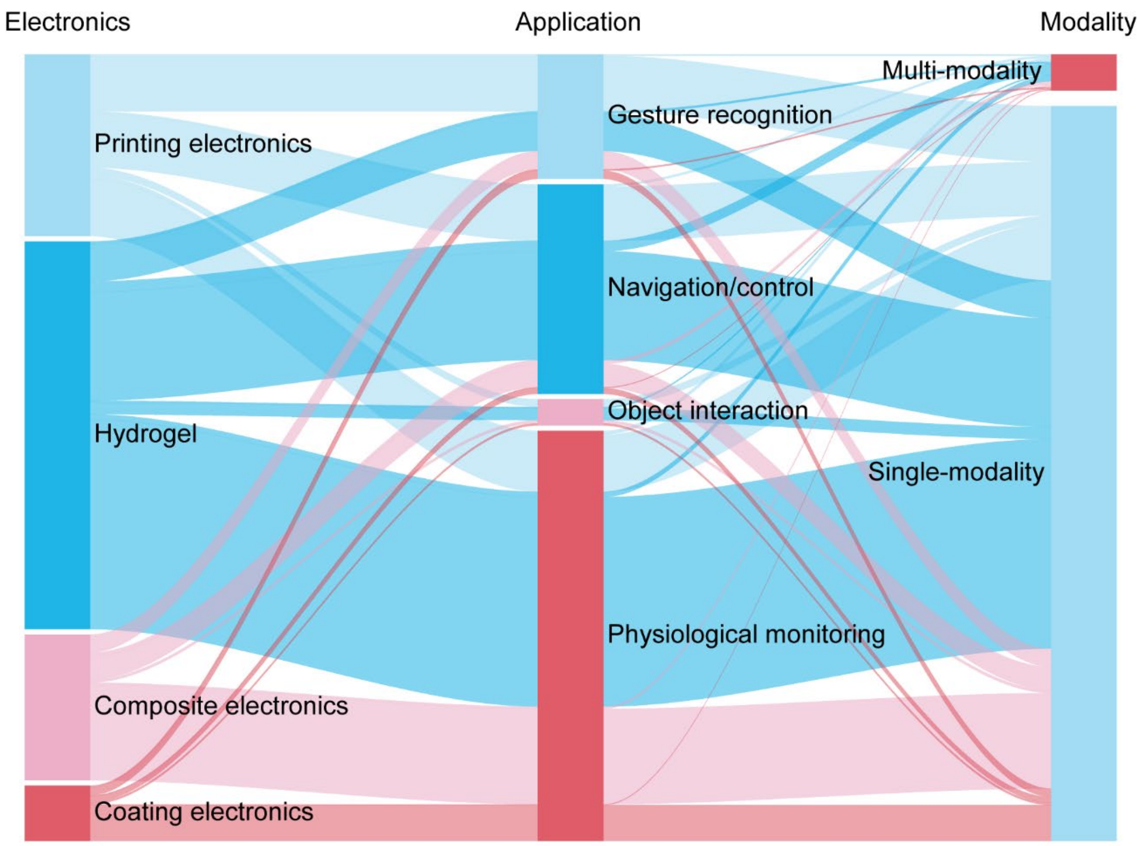


**Fig. S17. Sankey diagram for the applications and modality number of common electronics in research in the last three years.**

The statistical analysis was based on 1026 publications related to electronics over the past three years, sourced from the Web of Science database. A full allocation method was used here, ensuring that the total counts for each category at the first and second levels were identical, with no external attributions. The results highlight that electronics for intention interaction and information transmission accounted for a notable portion, while studies involving multi-modality research represent an even smaller fraction.

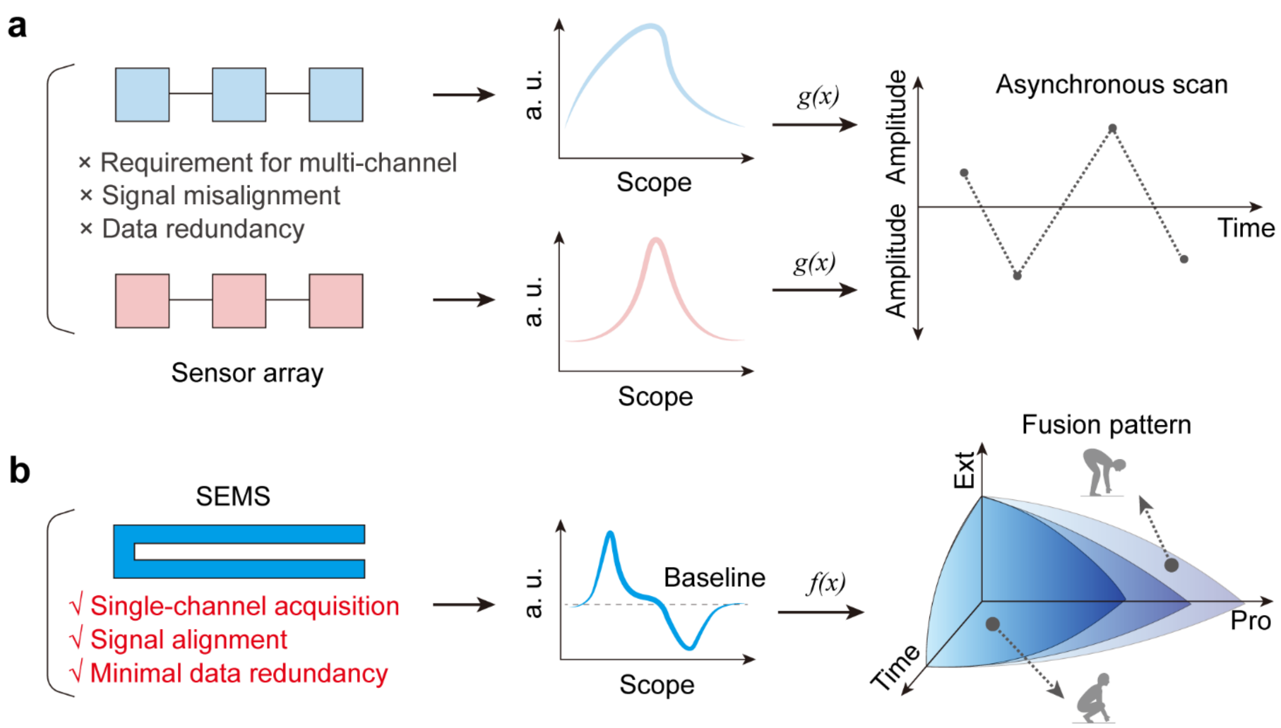


**Fig. S18. Comparison of traditional sensor arrays and the HDM metaskin for multi-modal sensing and data efficiency.**

(a) Limitations of traditional sensor arrays include the need for multi-channel acquisition, susceptibility to signal misalignment, and inherent data redundancy. These arrays produce localized outputs that are processed independently, resulting in constrained scope and diminished efficiency for multi-dimensional sensing tasks. (b) Advantages of the metaskin include single-channel acquisition, inherent signal alignment, and minimal data redundancy. By generating continuous and decoupled signals, the HDM metaskin seamlessly integrates proprioceptive (Pro) and exteroceptive (Ext) inputs. This integration enables three-dimensional mapping of sensing information, effectively bridging body posture and motion for precise contextual adaptation. These capabilities hold significant potential for advancing the understanding and reconstruction of complex body dynamics, facilitated by optimized device configurations and efficient interaction frameworks.

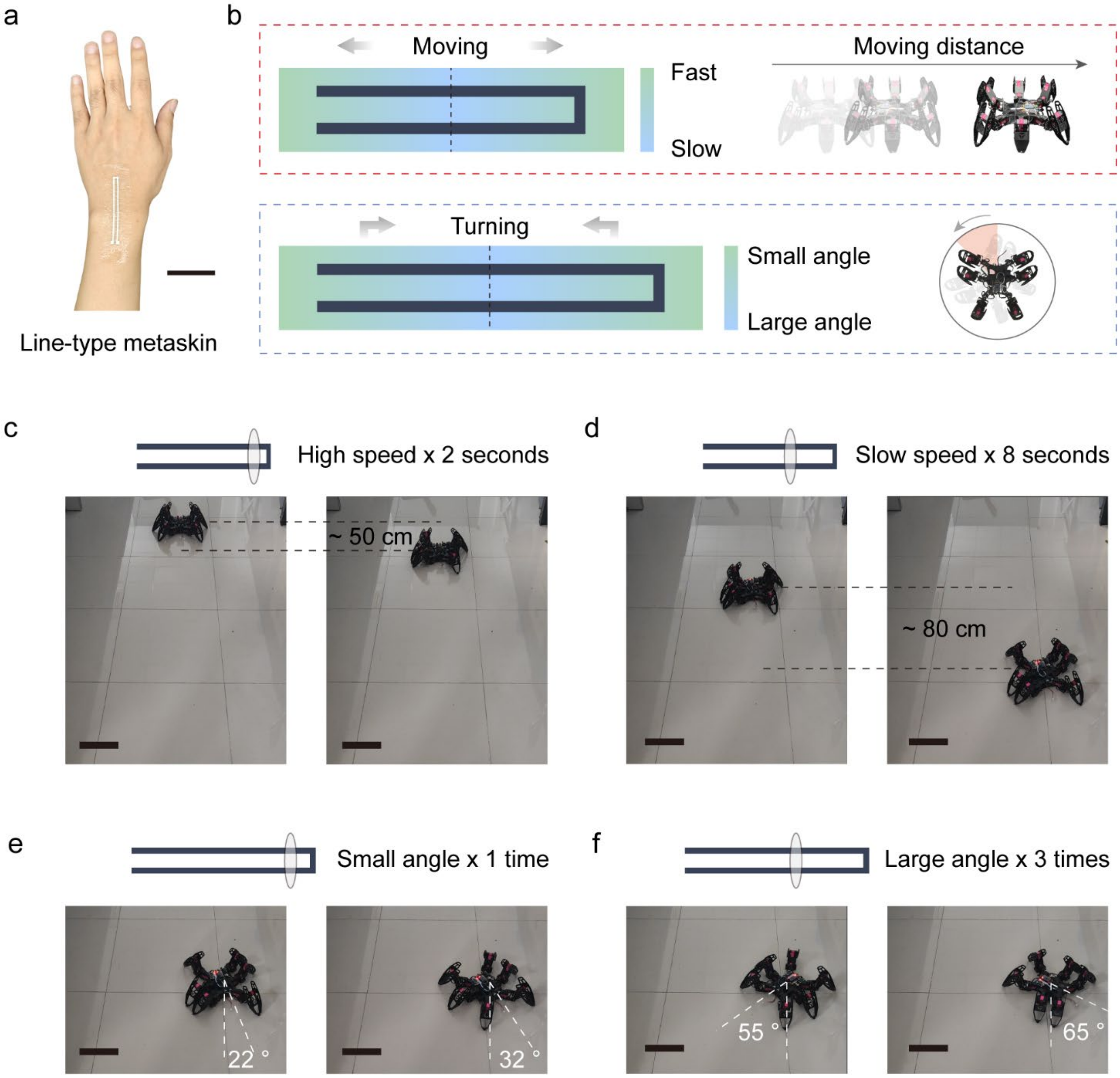


**Fig. S19 Hexapod robot precise navigation with a line-type HDM metaskin.**

(a) Morphology of the line-type HDM metaskin on the wrist (scale bar: 5 cm). (b) Control setup for the metaskin. The HDM metaskin allows different control modes depending on its stretching. In its original state, it functions as a moving context, where the touch position indicates the movement direction and speed. When stretched, it switches to a turning context, allowing precise steering angle adjustments. (c-f) Real-world scenes showing the hexapod robot's navigation, demonstrating accurate control of distance and angles. Scale bar: 20 cm.

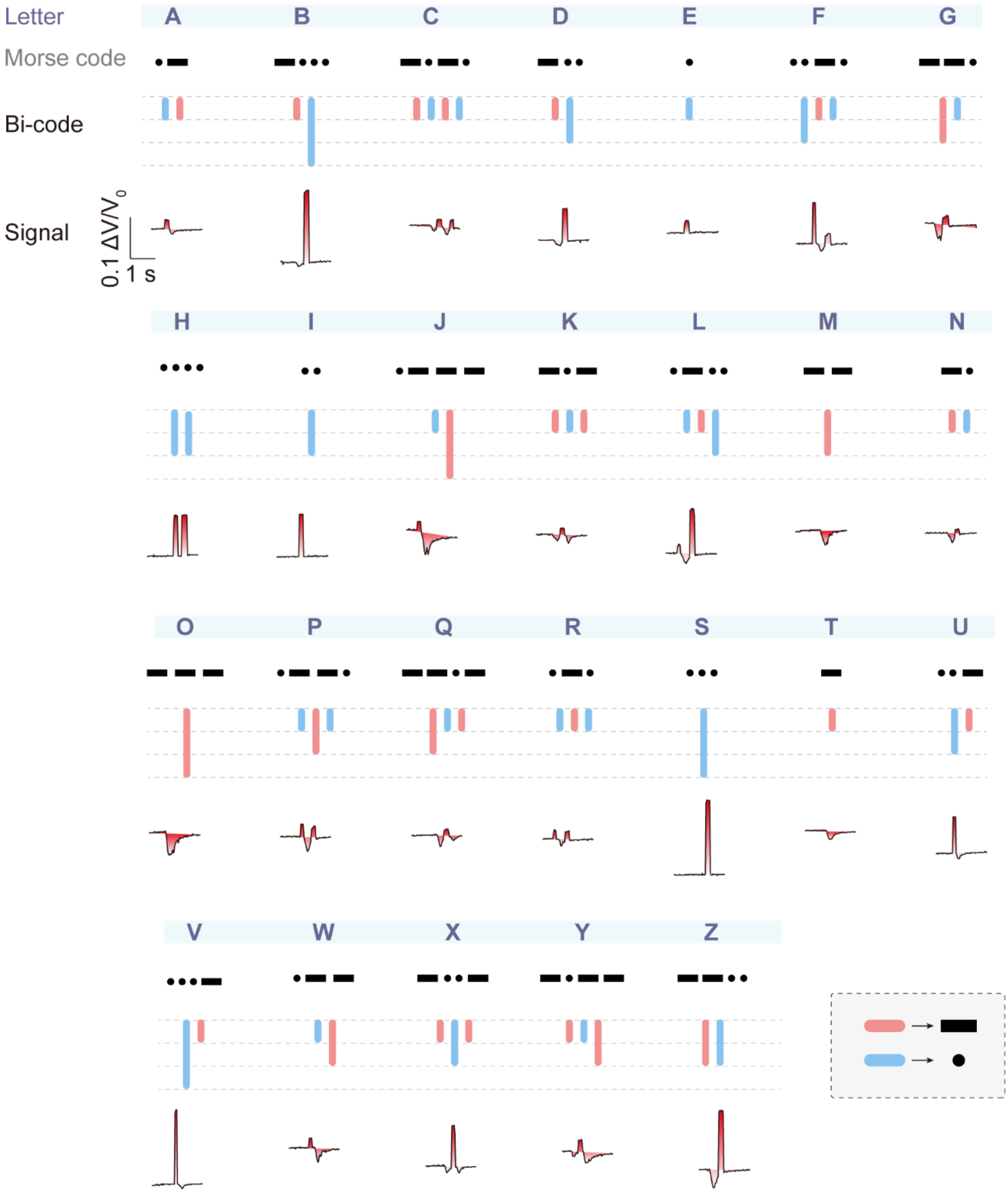


**Fig. S20. Correspondence between bi-coding, Morse code, and the 26 English letters, illustrating their mapping relationship and signal representation.**

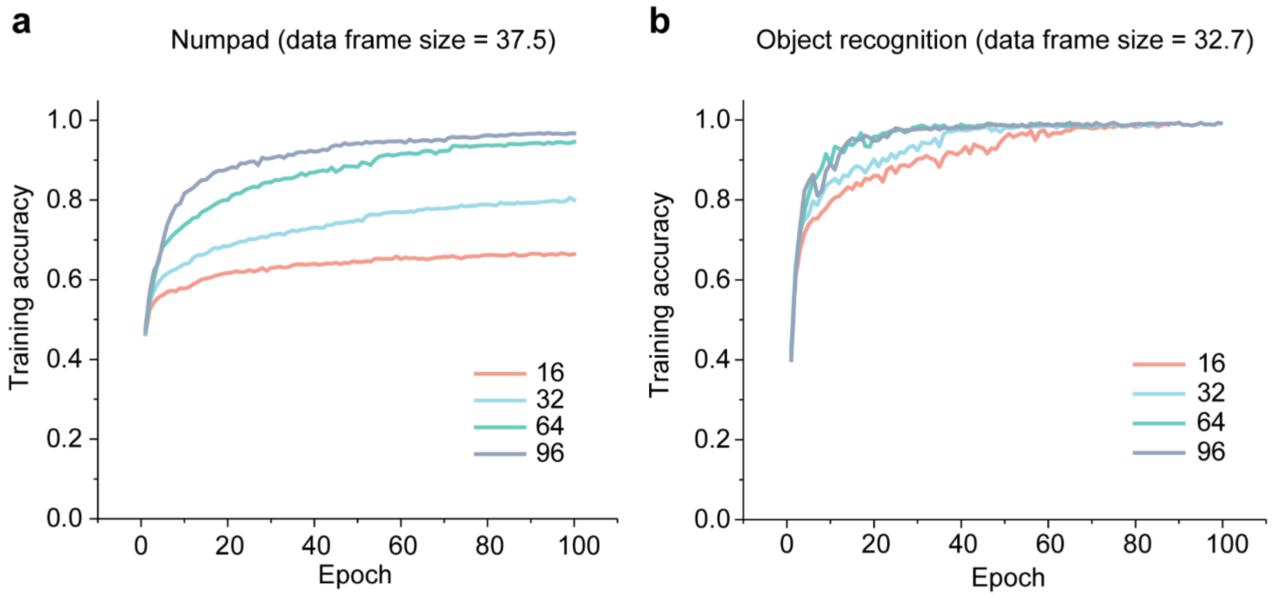


**Fig. S21 Performance comparison for different time window sizes.**

The training accuracy of (a) the numpad model and (b) the object recognition model using different time window sizes. Four models were trained with time window sizes of 16, 32, 64, and 96 using the same hand motion data. The results show that models trained with window sizes of 64 and 96 outperformed those trained with smaller window sizes, with the difference being particularly significant for the Numpad model. Since the typing action is single-triggered, a larger window size is expected to capture the complete data frame, leading to better performance. However, larger windows may also include signals from non-relevant numpad labels, causing the model to learn unnecessary features, despite performing well during training. In contrast, for the object recognition model, which is continuous-triggered, the impact of window size is relatively minor.

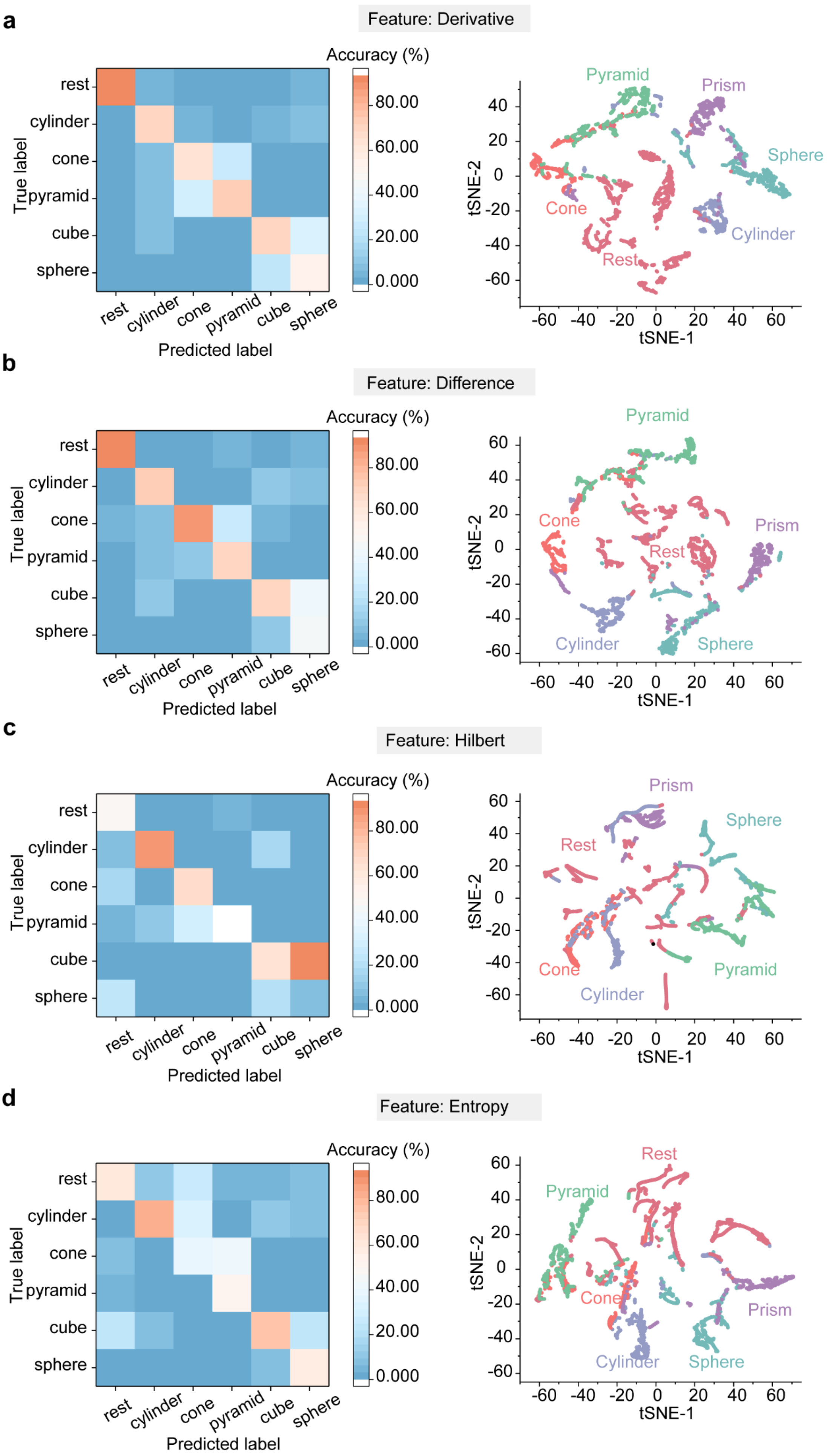
a
Feature: Derivative
b
Feature: Difference
c
Feature: Hilbert
d
Feature: Entropy
Accuracy (%)
80.00
60.00
40.00
20.00
0.000
True label
Predicted label
rest
cylinder
cone
pyramid
cube
sphere
tSNE-1
tSNE-2
Pyramid
Prism
Sphere
Cone
Cylinder
Rest

**Fig. S22 Performance comparison for models using different feature enhancement approaches.**

Confusion matrix and t-SNE visualization of models trained with data augmented using (a) derivatives, (b) differences, (c) Hilbert transform, and (d) entropy methods. Pre-training data treatment helps in extracting hidden features. Among the four data augmentation techniques, the model trained on the derivative-processed dataset shows superior performance, achieving the highest training accuracy. Additionally, the feature space demonstrates the most distinct separation in the t-SNE dimensionality reduction plot. The poor performance of models trained on the Hilbert-processed and entropy-processed datasets indicates that the cyclic and frequency characteristics in these datasets are weak.

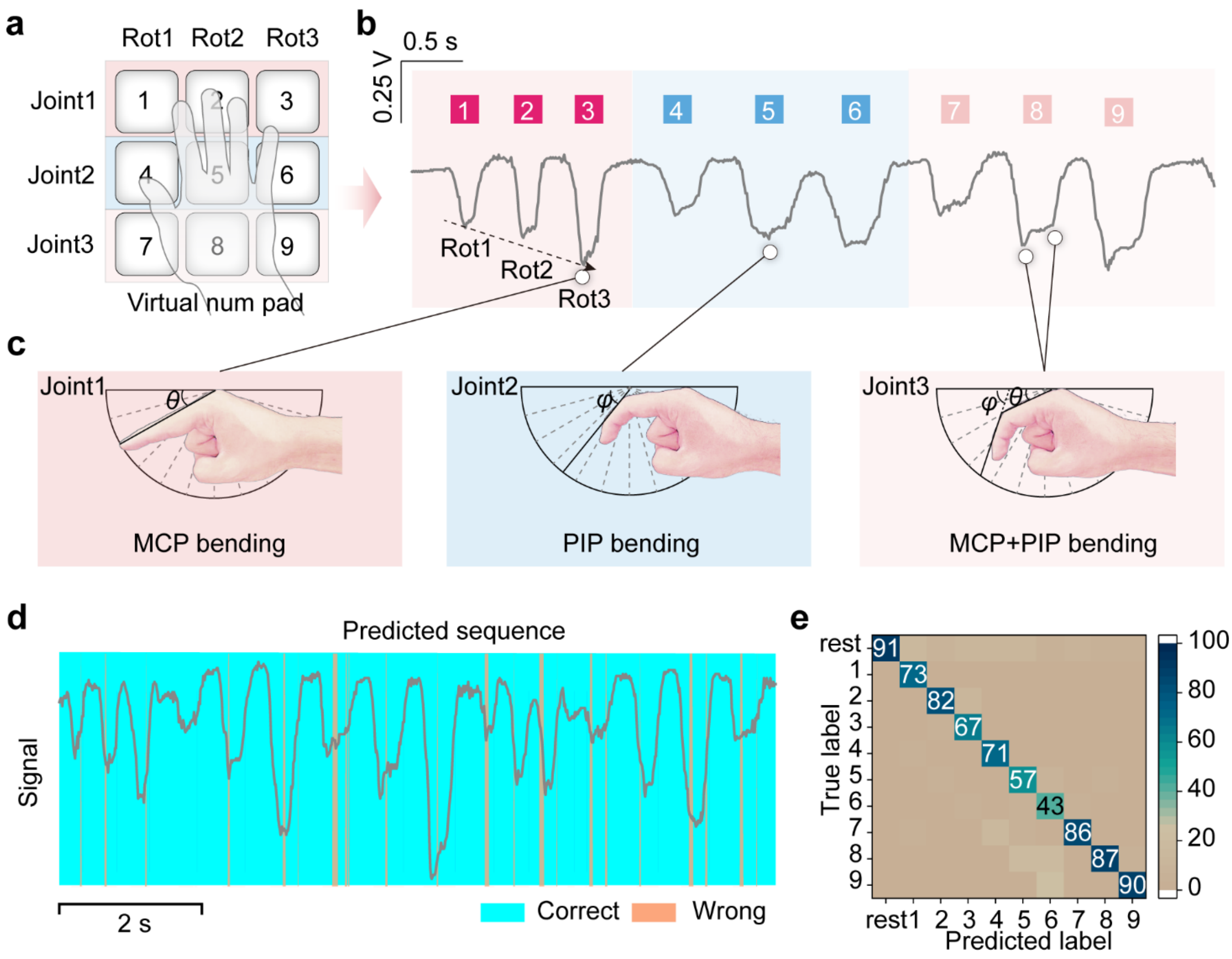


**Fig. S23. Virtual num pad interaction enabled by finger motion classification.**

(a) Spatial relationship between the virtual num pad and the hand. The numeric keypad is arranged in a nine-grid layout. The left-to-right numeric input is controlled by three types of motions (Joint1, Joint2, Joint3). Each joint motion is associated with a rotation angle (Rot1, Rot2, Rot3) to trigger specific numeric inputs. (b) Voltage signal pattern during sequential number input (1-9), demonstrating distinct signal peaks corresponding to joint rotations. (c) Illustration of three joint motions: MCP bending (Joint1), PIP bending (Joint2), and combined MCP+PIP bending (Joint3), in which θ and φ correspond to the bending angle of MCP and PIP, respectively. (d) Predicted signal sequence for continuous input, highlighting correct and incorrect predictions. (e) Confusion matrix of classification results, showing accuracy for each number input with rest state included.

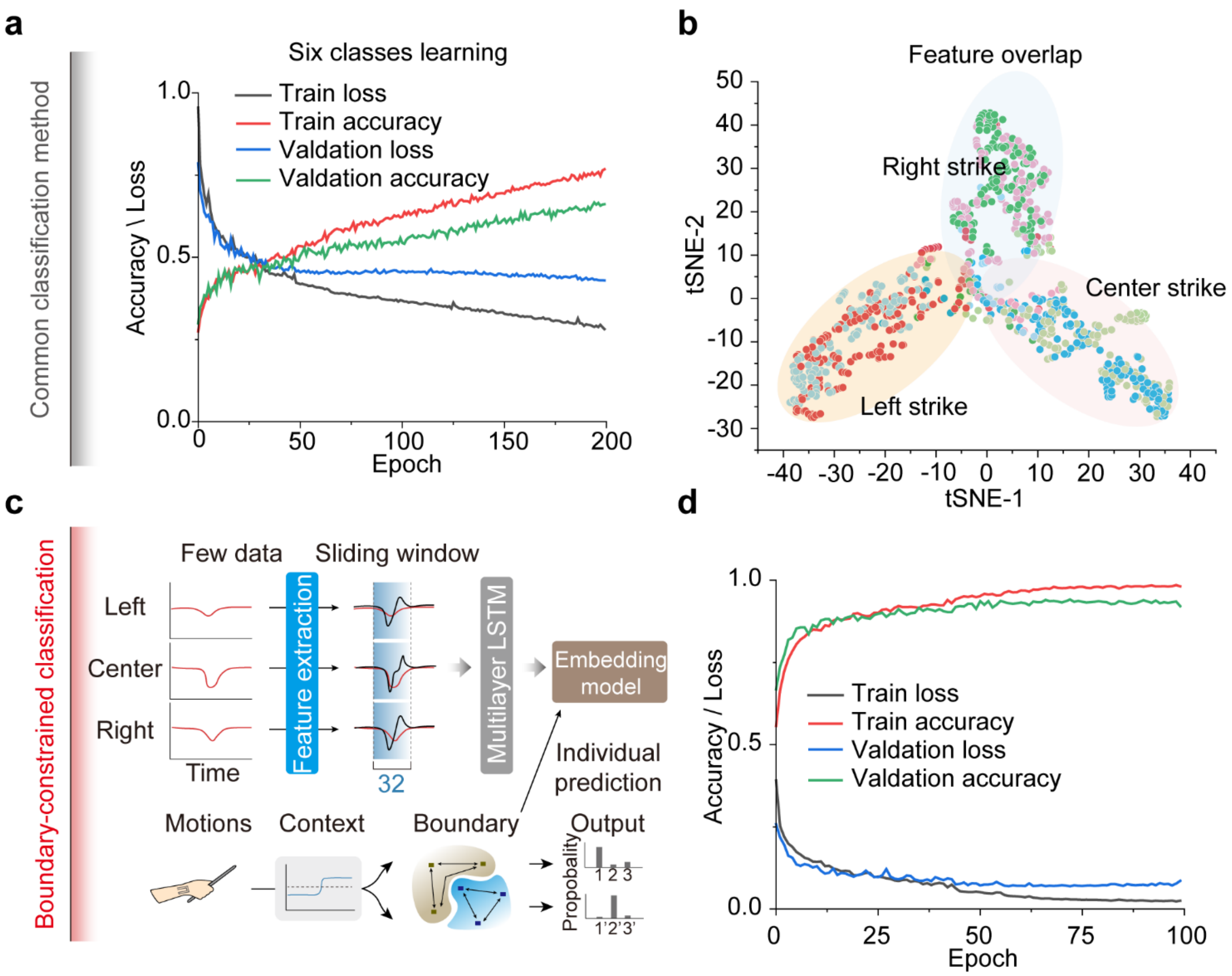


**Fig. S24. Implementation of seamless multi-scene interaction tasks integrating static postural features and dynamic motion features.**

(a) Training and validation curves for six-class learning (left, middle, and right wrist motions under two holding postures) using only proprioceptive signal input in the drum performance application. The lack of interaction between static postural features and dynamic motion features results in poor feature classification in machine learning tasks. Consequently, parallel mode control, combining proprioceptive motion and exteroceptive state, is often avoided in lightweight interaction designs due to its perceived unnaturalness. (b) t-SNE visualization of feature representation with proprioception-only input. Significant feature overlap between similar wrist motions demonstrates insufficient feature separation and classification capability. (c) Boundary-constrained classification method for contextual interaction tasks. The holding posture corresponds to the exteroceptive state and serves as a boundary condition to guide the machine learning model's output within the intended classification space, reducing training dimensionality and promoting feature separation. (d) Training and validation curves for three-class learning, demonstrating improved accuracy and feature discrimination. The trained model supports two distinct contexts, enabling robust multi-scene interaction.

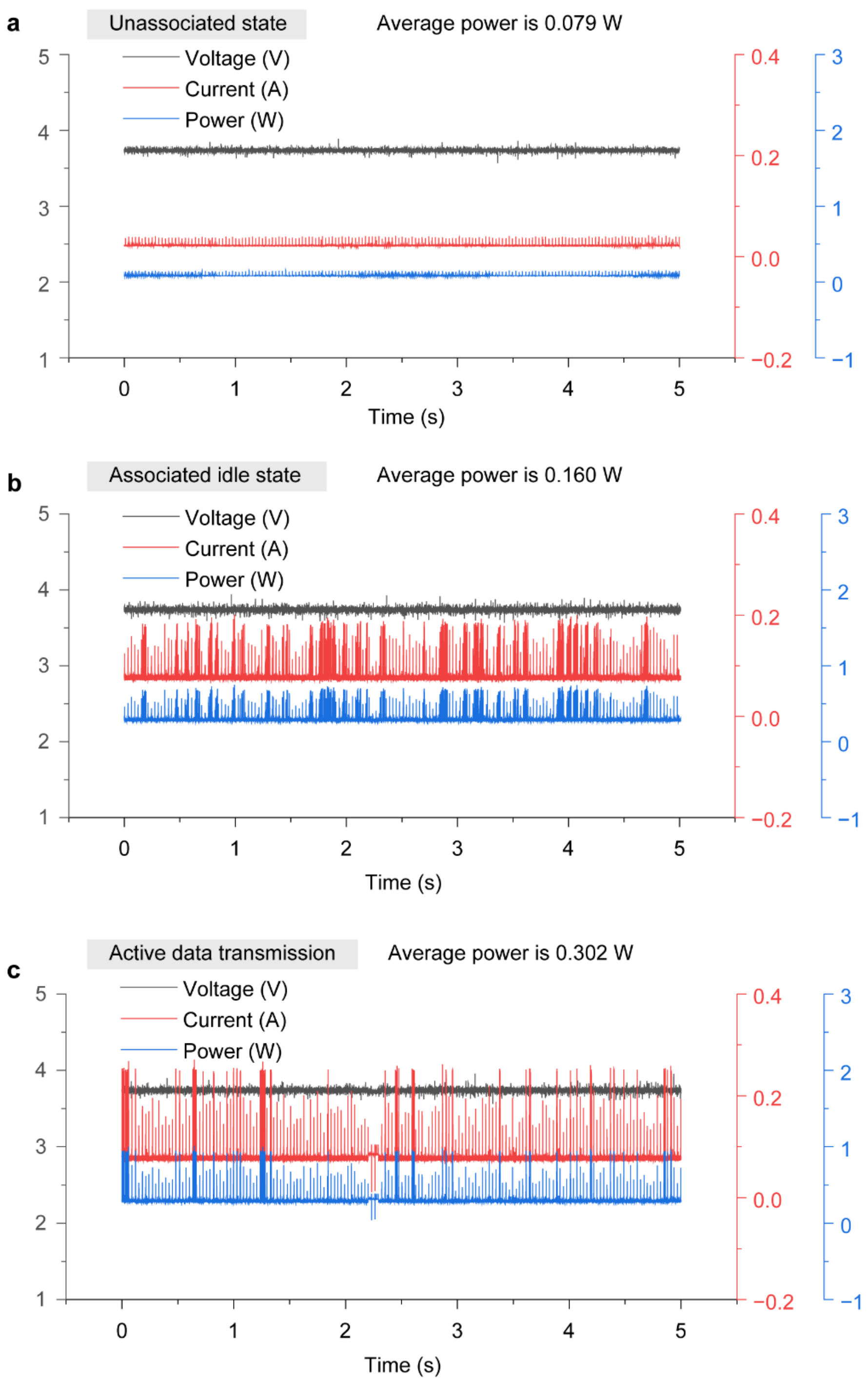


**Fig. S25. Power consumption of the portable sensor system.**

Working power consumption at three different connection states are recorded, including (a) unassociated state, (b) associated idle state, and (c) active data transmission. In the unassociated state, the power consumption (0.079 W) is primarily contributed by the microcontroller unit (MCU), analog-to-digital converter (ADC) modules, and the HDM sensor. When associated, the power consumption increased to 0.160 W and 0.302 W, respectively.
