# Supplementary material for: A hyperconformal dual-modal metaskin for well-defined and high-precision contextual interactions
Source: Nat Commun. 2025 Nov 26;16:10573. doi: 10.1038/s41467-025-65624-z (PMC12658143; doi:10.1038/s41467-025-65624-z)
Supplement: Supplementary file 2 — Description of Additional Supplementary Information [file 41467_2025_65624_MOESM2_ESM.pdf]

Description of Additional Supplementary Files

Supplementary Movie 1. Preparation of the HDM metaskin and transfer to skin.

Supplementary Movie 2. Contextual interaction with a hexapod robot.

Supplementary Movie 3. Contextual interaction with a hexapod robot: implementing sophisticated commands.

Supplementary Movie 4. Bi-coding for typing.

Supplementary Movie 5. Bi-coding for mouse control.

Supplementary Movie 6. Object-based multi-scenario drumming interaction.

Supplementary Movie 7. Wireless transmission of sensing signals.
