# Supplementary material for: A hyperconformal dual-modal metaskin for well-defined and high-precision contextual interactions
Source: Nat Commun. 2025 Nov 26;16:10573. doi: 10.1038/s41467-025-65624-z (PMC12658143; doi:10.1038/s41467-025-65624-z)
Supplement: Supplementary file 10 — Reporting Summary [file 41467_2025_65624_MOESM10_ESM.pdf]

nature portfolio

Corresponding author(s): Xinqin Liao

Last updated by author(s): 2025-9-8

Reporting Summary

Nature Portfolio wishes to improve the reproducibility of the work that we publish. This form provides structure for consistency and transparency in reporting. For further information on Nature Portfolio policies, see our Editorial Policies and the Editorial Policy Checklist.

Statistics

For all statistical analyses, confirm that the following items are present in the figure legend, table legend, main text, or Methods section.

n/a	Confirmed	
☐	☒	The exact sample size (*n*) for each experimental group/condition, given as a discrete number and unit of measurement
☐	☒	A statement on whether measurements were taken from distinct samples or whether the same sample was measured repeatedly
☒	☐	The statistical test(s) used AND whether they are one- or two-sided<br>*Only common tests should be described solely by name; describe more complex techniques in the Methods section.*
☒	☐	A description of all covariates tested
☒	☐	A description of any assumptions or corrections, such as tests of normality and adjustment for multiple comparisons
☐	☒	A full description of the statistical parameters including central tendency (e.g. means) or other basic estimates (e.g. regression coefficient) AND variation (e.g. standard deviation) or associated estimates of uncertainty (e.g. confidence intervals)
☒	☐	For null hypothesis testing, the test statistic (e.g. *F*, *t*, *r*) with confidence intervals, effect sizes, degrees of freedom and *P* value noted<br>*Give P values as exact values whenever suitable.*
☒	☐	For Bayesian analysis, information on the choice of priors and Markov chain Monte Carlo settings
☒	☐	For hierarchical and complex designs, identification of the appropriate level for tests and full reporting of outcomes
☒	☐	Estimates of effect sizes (e.g. Cohen's *d*, Pearson's *r*), indicating how they were calculated

Our web collection on statistics for biologists contains articles on many of the points above.

Software and code

Policy information about availability of computer code

Data collection	The data for application and training were collected using custom Python code (Python 3.9), while other data were acquired using the proprietary instrument-integrated software provided by the commercial systems, as described in the Methods section. These software packages are not publicly available outside the instruments. The custom code is available from https://github.com/yueohuim/CNN-LSTM-for-finger-movement-recognition.
Data analysis	We performed all machine learning procedures using Python software (Python 3.9). Data analysis was implemented using the commercial software Matlab (version R2022b) and Origin (version 2017). The simulation was implemented using the commercial software COMSOL 5.6.

For manuscripts utilizing custom algorithms or software that are central to the research but not yet described in published literature, software must be made available to editors and reviewers. We strongly encourage code deposition in a community repository (e.g. GitHub). See the Nature Portfolio guidelines for submitting code & software for further information.

Data

Policy information about availability of data

All manuscripts must include a data availability statement. This statement should provide the following information, where applicable:

- Accession codes, unique identifiers, or web links for publicly available datasets
- A description of any restrictions on data availability
- For clinical datasets or third party data, please ensure that the statement adheres to our policy

The data support the findings of this study are available within the main text and the Supplementary Information/Source Data file. The raw training data for machine

learning are included in the provided code. Source data are provided with this paper.

Research involving human participants, their data, or biological material

Policy information about studies with human participants or human data. See also policy information about sex, gender (identity/presentation), and sexual orientation and race, ethnicity and racism.

Reporting on sex and gender	Motion signal acquisition experiments were performed on a male participant, as gender-specific effects were deemed irrelevant to the study conclusions.
Reporting on race, ethnicity, or other socially relevant groupings	This work did not require relevant review.
Population characteristics	No demographic data collected.
Recruitment	No participants recruited.
Ethics oversight	All procedures involving human research participants were conducted in accordance with the experimental protocol approved by the Ethics Committee of Xiamen University (XDYX202501K02).

Note that full information on the approval of the study protocol must also be provided in the manuscript.

Field-specific reporting

Please select the one below that is the best fit for your research. If you are not sure, read the appropriate sections before making your selection.

[x] Life sciences [ ] Behavioural & social sciences [ ] Ecological, evolutionary & environmental sciences

For a reference copy of the document with all sections, see nature.com/documents/nr-reporting-summary-flat.pdf

Life sciences study design

All studies must disclose on these points even when the disclosure is negative.

Sample size	Sample sizes for error analysis were determined based on the minimum requirements derived from error analysis (n=3~6). Training data volumes were optimized under the principle of few-shot learning while ensuring model convergence. No formal statistical method was used to predetermine sample sizes. Sample sizes were chosen based on established practices in experimental feasibility and prior experience. They are considered sufficient for reliable interpretation of results.
Data exclusions	No data were excluded from the analysis.
Replication	All manufacturing method and result measures were repeated over 3 times for reproducibility and the results were all successful.
Randomization	As all specimens originated from the same fabrication batch, potential confounding factors related to batch-to-batch variations were inherently controlled in this study.
Blinding	Due to fully automated measurement protocols, blinding was deemed unnecessary as no subjective judgment was involved in data collection.

Reporting for specific materials, systems and methods

We require information from authors about some types of materials, experimental systems and methods used in many studies. Here, indicate whether each material, system or method listed is relevant to your study. If you are not sure if a list item applies to your research, read the appropriate section before selecting a response.

Materials & experimental systems

n/a	Involved in the study	
[x]	[ ]	Antibodies
[x]	[ ]	Eukaryotic cell lines
[x]	[ ]	Palaeontology and archaeology
[x]	[ ]	Animals and other organisms
[x]	[ ]	Clinical data
[x]	[ ]	Dual use research of concern
[x]	[ ]	Plants

Methods

n/a	Involved in the study	
[x]	[ ]	ChIP-seq
[x]	[ ]	Flow cytometry
[x]	[ ]	MRI-based neuroimaging

Plants

Seed stocks	Not applicable
Novel plant genotypes	Not applicable
Authentication	Not applicable
